# Awareness and knowledge of dental erosion and its association with beverage consumption: a multidisciplinary survey

**DOI:** 10.1186/s12903-022-02065-w

**Published:** 2022-02-11

**Authors:** Jeremiah Schmidt, Boyen Huang

**Affiliations:** 1 School of Dentistry and Health Sciences, Charles Sturt University, Orange, NSW Australia; 2 Department of Primary Dental Care, University of Minnesota School of Dentistry, 515 Delaware St SE, Minneapolis, MN 55455 USA

**Keywords:** Tooth erosion, Dental erosion, Tooth wear, Beverage consumption, Awareness, Health literacy, University students

## Abstract

**Background:**

Erosive tooth wear has significant impacts on oral and general health. This study aimed to measure the awareness of dental erosion to establish the relationships among sociodemographic factors, awareness and knowledge of dental erosion, and beverage consumption behaviours, in a sample of university students in Australia.

**Methods:**

All undergraduate students enrolled in the targeted programs at an Australian University were invited. A total of 418 students consented to participate. Each was assessed with an online questionnaire.

**Results:**

The awareness rate of dental erosion was 92.1%. Soft drinks and fruit juice were most often perceived as acidic beverages by the respondents. The students of greater age, and studying pharmacy, paramedicine, physiotherapy or science, were less likely to be aware of dental erosion. Those students that were aware of dental erosion also had better knowledge of dental erosion, which was associated with a reduced quantity of daily acidic beverage consumption as well. The students that predominantly resided overseas had less knowledge of dental erosion. The students that were of greater age, that were studying clinical science, pharmacy, medical science, paramedicine, or science, and that correctly identified more types of acidic beverages, more often consumed acidic beverages in high-risk patterns.

**Conclusions:**

Erosive tooth wear is a relevant matter in dentistry, nutrition and public health. Within a university setting, the effect of education on oral health literacy and beverage consumption behaviour is confirmed. Dental and health professionals should actively educate the individuals and communities about dental erosion and motivate them to change acidic beverage consumption behaviours.

## Background

Dental erosion is a process of the loss of dental hard tissue caused by chemical dissolution without the involvement of oral bacteria [1, 2]. Acids and specific chemicals can erode the surface of teeth and dental restorations and then lead to structure loss [1, 3, 4]. When dental erosion is the principal cause of tooth substance loss, the condition is defined as erosive tooth wear [2] that generally presents as lesions with a smooth melted appearance of enamel and dentine [5]. Whilst the published erosive tooth wear studies on adults are outnumbered by those on children and adolescents [6], the prevalence in adults widely ranges from 2% [7] to 100% [8] and the percentage increases with age [9, 10]. Potential consequences of dental erosion include dentine hypersensitivity, dental pain, unsatisfactory dental esthetics, and/or impaired oral function [11]. In addition to the preventive and restorative approaches provided by dental professionals to manage dental erosion and its clinical consequences [11], some self-care measures to prevent dental erosion have been suggested [12, 13]. These self-care methods are developed to reduce the exposure of the dentition to intrinsic gastrointestinal and extrinsic dietary acids which have been considered as the aetiological factors of dental erosion [14]. Particularly, acidic beverages such as soft drinks, sports drinks, energy drinks, juices, iced tea, and sparkling water show a potential to cause dental erosion [15, 16]. Erosive tooth wear not only is a dental disorder but also requires interprofessional attention from physicians [17–19], nurses [19, 20], dietitians [21], pharmacists [19], and mental health professionals [19, 22].

With a PubMed and Google Scholar search using a combination of the terms of “awareness”, “knowledge”, “understanding”, “dental erosion” and “erosive wear”, and a critical review, six papers using participants of 18 years or older, providing clear results, written in English and published before 2018 were identified [23–28]. With an awareness rate ranging from 30% [25] to 97% [27], a simple average of the six diverse groups showed only 57.4% of the participants had heard of dental erosion or erosive tooth wear [23–28]. Further, not only laypersons but also some dentists and dental students have shown a lack of knowledge of dental erosion [26–28]. To empower an individual’s ability to improve their oral and general wellbeing, having some level of oral health literacy is essential [29]. There has been evidence to support a relationship between higher oral health literacy and better dental health [30] as well as a positive association between greater health awareness and healthy dental behaviours [31]. Conversely, lower awareness of the impact acidic drinks have on the aetiology of dental erosion is associated with an increase in the frequency acidic beverages are consumed, and consequently related to the individual's risk of dental erosion [24]. Identifying the factors that contribute to health behaviours facilitates more targeted approaches to increase the awareness and knowledge of oral conditions and improve health promotion [32]. Thus, it is relevant to enhance awareness and knowledge of dental erosion in individuals and communities and thereby achieve effective prevention of the disease and offer a better prognosis for the dental hard tissues and any restorative treatment.

With the high prevalence of erosive tooth wear internationally [8, 9], correlations with general health [14, 17–22], and known impacts on oral health [11], erosive tooth wear has become a developing issue in public health and a challenge for the century [1]. Nevertheless, the reported levels of awareness and knowledge of dental erosion among earlier studies varied greatly and there was a lack of Australian data. Moreover, the factors contributing to the awareness and knowledge of dental erosion remained unclear and there has been no literature on its association with acidic beverage consumption. Therefore, this study aimed to measure the awareness and knowledge of dental erosion in a sample of university undergraduate students in Australia. A special interest was to investigate the relationships between awareness and knowledge of dental erosion, beverage consumption behaviours, and respondent's sociodemographic factors including age, academic field, and geographic remoteness, with a purpose to facilitate health promotion actions on prevention of erosive tooth wear, which could benefit a broader target population.

## Methods

### Study design

A cross-sectional investigation was conducted in August 2018. Before the commencement of this study, ethics approval was granted by the Human Research Ethics Committee of an Australian university (Protocol Number: H18156). The population included all students enrolled in the Bachelor programs of clinical science, dentistry, medical science, oral health (dental hygiene and therapy), pharmacy, physiotherapy, paramedicine, and science at an Australian university (N ≊ 1,000, not a definite number as student withdrawal and late enrolment occurred in August). For reporting 95% confidence limits for the awareness rate of dental erosion with satisfactory precision, the minimum sample size (n) required was 273. This calculation was made with an expected awareness rate at 57.4% (the mean awareness rate of the 6 papers reviewed) [23–28] and an acceptable margin of error at 5%, using Epi Info 7 (Centers for Disease Control and Prevention, Atlanta, GA, USA).

### Participant selection

As the response rate of an online survey was unpredictable and the population size at the university was not too large to manage, in order to obtain the minimum sample size required, the entire population of interest was studied. This method, described as ‘total population sampling’ in literature, can be used when a potential sample size is contingent on the response rate [33]. All students from all year levels of the targeted programs, aged 18 or over, and with working proficiency in English were invited to participate. Each was sent an electronic web address to an online, self-administered questionnaire hosted by Survey Monkey via their respective online university learning platforms by their subject coordinators and through private Facebook groups created and administrated by the members of different degrees and cohorts. Duplicate responses were prevented by the Survey Monkey software using their inbuilt ‘Collector Options’ that involve browser tracking. The tracking data cannot be used to identify any individuals and are not disclosed to the researchers. A chance to win an iPad was offered as the reward for participation. Upon opening of the online questionnaire, the respondent was presented with a digital copy of the participant information statement. Consent for the use and interpretation of data was implied upon submitting a response to the online questionnaire as outlined in the participant information statement.

### Questionnaire design

The questionnaire was composed of 13 items adopted from previous publications [26–28, 34]. The data collected via the questionnaire included age (by subtracting the birth year from 2018), academic field, geographic remoteness, awareness and knowledge of dental erosion and acidic beverages, and beverage consumption behaviours. Geographic remoteness was measured by manipulating the postcode to the Australian Statistical Geography Standard (ASGS) Remoteness Structure [35] to classify respondents based on predominantly residing in major cities, regional/remote or overseas settings while not attending university. Awareness was evaluated with the question (3) ‘have you heard of the term dental erosion in relation to dental health?’. Knowledge of dental erosion was determined with a knowledge score (disease-specific) and an identified acidic beverages (ID) score (nutrition-specific). Participants were given a knowledge score of 0 to 3 out of 3 as a numerical representation of the number of correct ‘knowledge’ questions regarding dental erosion. The three ‘knowledge’ questions used were (1) ‘if a drink (e.g. orange juice) has no added sugar listed on the packaging, will it be possible for it to harm your teeth?’, (6) ‘would you recommend reducing sugar intake to reduce dental erosion?’, and (9) ‘which of the following would cause dental erosion?’. The ID score of 0 to 11 out of 11 was defined as the number of correctly identified acidic beverages listed in the questionnaire as determined from the published literature [15, 16]. The 11 acidic beverages were soft drink, fruit juice, sugar-free soft drink, energy drink, vitamin water, sparkling water, sports drink, iced tea, wine, premixed alcoholic drink, and beer. Beverage consumption data included the number of preferred beverage types (different acidic beverages consumed multiple times per week), quantity of beverage consumption (how many 250 ml cups consumed per day, provided with a volume guide of commercial beverages), and beverage consumption patterns. The beverage consumption patterns were classified as high-risk patterns (between meals or sip over a period longer than 10 min) and low-risk patterns (with food, sip quickly or drink mainly water) [36]. Except for the item of the birth year, all other 12 items used predetermined answers other than open-ended questions to yield a higher response rate [37].

### Data collection

A pilot survey was administered to 10 lay respondents to verify the structure, accessibility, and comprehension of the questionnaire. After this was deemed successful, the data of the pilot study were destroyed prior to the distribution of the survey link to the 1000 students. ‘Collector Options’ within Survey Monkey were utilised to prevent multiple responses from the same respondent and/or returning to previous questions.

### Data analysis

Data conversion and statistical analysis were carried out with IBM SPSS Statistics 27.0 (IBM, Chicago, IL, USA). Data analysis included descriptive statistics (frequency distribution and cross-tabulation). To achieve the objectives and interests of this study, the statistical analysis was structured in three steps. The first step was to examine the influence of sociodemographic variables on awareness. The second step focused on characterising the knowledge with the contribution of awareness as well. Following these, the effects on beverage consumption behaviours, from the awareness and knowledge of dental erosion as well as the sociodemographic variables, were evaluated. The awareness of dental erosion and beverage consumption patterns were assessed with a backward-conditional binary logistic regression method. The goodness of fit for the logistic regression model was determined with the Hosmer and Lemeshow test. The knowledge score, ID score, number of preferred beverage types, and quantity of beverage consumption were examined with a backward linear regression approach. Dummy variables were created to represent individual categories of the polytomous variables such as academic field and geographic remoteness in the linear regression models. Multivariate analysis was performed to evaluate the contribution of the variables [38]. The significance level was set at 5%.

## Results

### Description of the study sample

Of the 1000 students invited to complete the survey, 434 accessed the survey link and 421 of them completed the questionnaire. This contributed to a response rate of 42.1%. Three respondents with incomplete data were excluded from statistical analysis and consequently, 418 subjects were included in the final sample, leading to a completion rate of 96.3%. The participants’ age ranged from 18 to 72 years, with a median age of 23 years and a mean age of 25.7 ± 7.8 years. Of the respondents, 126 (30.1%) were enrolled in a dental field (dentistry or oral health) while 292 (69.9%) were studying in other academic fields. The students predominantly resided in Australian regional and remote areas (57.6%) followed by Australian major cities (39.5%) and overseas (2.9%). Table 1 shows the frequency distribution of the participants’ sociodemographic data.

**Table 1:** Awareness of dental erosion by age, academic field and geographic remoteness (n = 418)

	Aware [n (%)]	Not aware [n (%)]	All [n (%)]	Unadjusted^a^ OR (95% CI)	*p* value	Adjusted^b^ OR (95% CI)	*p* value
*Age in years*^c^							
18–20	85 (93.4)	6 (6.6)	91 (21.8)	0.927 (0.897, 0.959)	< 0.001*	0.933 (0.896, 0.971)	0.001*
21–30	241 (95.3)	12 (4.7)	253 (60.5)				
31–40	39 (84.8)	7 (15.2)	46 (11.0)				
41–72	20 (71.4)	8 (28.6)	28 (6.7)				
*Academic field*							
Dentistry	104 (99.0)	1 (1.0)	105 (25.1)	1		1	
Oral Health	21 (100.0)	0 (0)	21 (5.0)	15,533,411.95 (0, ∞)	0.998	15,769,075.71 (0, ∞)	0.998
Clinical Science	31 (96.9)	1 (3.1)	32 (7.7)	0.298 (0.018, 4.905)	0.397	0.338 (0.020, 5.638)	0.450
Pharmacy	18 (90.0)	2 (10.0)	20 (4.8)	0.087 (0.007, 1.005)	0.050	0.080 (0.007, 0.934)	0.044*
Medical Science	48 (90.6)	5 (9.4)	53 (12.7)	0.092 (0.010, 0.812)	0.032*	0.205 (0.021, 1.984)	0.171
Paramedicine	29 (85.3)	5 (14.7)	34 (8.1)	0.056 (0.006, 0.496)	0.010*	0.060 (0.007, 0.534)	0.012*
Physiotherapy	96 (90.6)	10 (9.4)	106 (25.4)	0.092 (0.012, 0.735)	0.024*	0.098 (0.012, 0.782)	0.028*
Science	38 (80.9)	9 (19.1)	47 (11.2)	0.041 (0.005, 0.331)	0.003*	0.087 (0.010, 0.763)	0.028*
*Geographic remoteness*							
Australian major city	152 (92.1)	13 (7.9)	165 (39.5)	1			
Australian regional and remote	223 (92.5)	18 (7.5)	241 (57.6)	1.060 (0.504, 2.227)	0.879		0.309
Overseas	10 (83.3)	2 (16.7)	12 (2.9)	0.428 (0.085, 2.162)	0.304		0.355

*p < 0.05

^a^Odds ratio in the univariate regression model

^b^Odds ratio in the multivariate regression model

^c^Age calculated as years in statistical analysis—age ranges presented in the table to reduce the table length

### Awareness of dental erosion

A total of 385 participants (92.1%) showed the awareness of dental erosion. The students that were of greater age (*p* = 0.001, OR = 0.933, 95% CI: 0.896, 0.971) and that were studying pharmacy (*p* = 0.044, OR = 0.080, 95% CI: 0.007, 0.934), paramedicine (*p* = 0.012, OR = 0.060, 95% CI: 0.007, 0.534), physiotherapy (*p* = 0.028, OR = 0.098, 95% CI: 0.012, 0.782) or science (*p* = 0.028, OR = 0.087, 95% CI: 0.010, 0.763) were less likely to be aware of dental erosion. Other academic fields and geographic remoteness were not related to the awareness of dental erosion (*p* ≥ 0.171) (Table 1). The Hosmer–Lemeshow test did not identify any differences between the expected and observed proportions (*p* = 0.543, χ^2^ = 6.946, df = 8) and this indicated a good fit for the logistic regression model.

### Knowledge of dental erosion

The knowledge score of dental erosion in this sample ranged from 0 to 3 points, with a mean of 1.2 ± 0.7 points and a median of 1 point. Table 2 shows the frequency distribution of the knowledge score by age, academic field, geographic remoteness, and awareness of dental erosion. Based on the multivariate linear regression model, the knowledge score was higher among those students who enrolled in dentistry (*p* < 0.001, B = 0.863, 95% CI: 0.746, 0.980) or oral health (*p* < 0.001, B = 0.420, 95% CI: 0.196, 0.645), and that were aware of dental erosion (*p* < 0.001, B = 0.983, 95% CI: 0.796, 1.170). The students that predominantly resided overseas had a lower knowledge score of dental erosion (*p* = 0.006, B = − 0.420. 95% CI: − 0.717, − 0.124). Age, other academic fields, and other geographic areas were not related to the knowledge score (*p* ≥ 0.073) (Table 2).

**Table 2:** Knowledge score of dental erosion by age, academic field and geographic remoteness (n = 418)

	Knowledge score of dental erosion [n (%)]				Unadjusted^a^ B coefficient	*p* value	Adjusted^b^ B coefficient	*p* value
	0	1	2	3				
*Age in years*^c^								
18–20	6 (6.6)	68 (74.7)	12 (13.2)	5 (5.5)	− 0.012 (− 0.021, − 0.004)	0.004*	0.006 (− 0.001, 0.012)	0.095
21–30	14 (5.5)	175 (69.2)	40 (15.8)	24 (9.5)				
31–40	7 (15.2)	32 (69.6)	3 (6.5)	4 (8.7)				
41–72	5 (17.9)	22 (78.6)	1 (3.6)	0 (0)				
*Academic field*								
Dentistry	1 (1.0)	40 (38.1)	35 (33.3)	29 (27.6)	0.883 (0.754, 1.011)	< 0.001*	0.863 (0.746, 0.980)	< 0.001*
Oral Health	0 (0)	15 (71.5)	2 (9.5)	4 (19.0)	0.275 (− 0.030, 0.579)	0.071	0.420 (0.196, 0.645)	< 0.001*
Clinical Science	2 (6.3)	28 (87.4)	2 (6.3)	0 (0)	− 0.233 (− 0.483, 0.017)	0.068		0.861
Pharmacy	2 (10.0)	18 (90.0)	0 (0)	0 (0)	− 0.331 (− 0.642, − 0.020)	0.037*		0.634
Medical Science	4 (7.5)	48 (90.6)	1 (1.9)	0 (0)	− 0.311 (− 0.510, − 0.113)	0.002*		0.292
Paramedicine	6 (17.6)	28 (82.4)	0 (0)	0 (0)	− 0.426 (− 0.667, − 0.186)	0.001*		0.250
Physiotherapy	8 (7.5)	87 (82.1)	11 (10.4)	0 (0)	− 0.251 (− 0.402, − 0.099)	0.001*		0.073
Science	9 (19.2)	33 (70.2)	5 (10.6)	0 (0)	− 0.338 (− 0.547, − 0.130)	0.002*		0.794
*Geographic remoteness*								
Australian major city	14 (8.5)	104 (63.0)	28 (17.0)	19 (11.5)	0.165 (0.029, 0.301)	0.017*		0.192
Australian regional & Remote	15 (6.2)	187 (77.6)	26 (10.8)	13 (5.4)	− 0.146 (− 0.280, − 0.012)	0.033*		0.192
Overseas	3 (25.0)	6 (50.0)	2 (16.7)	1 (8.3)	− 0.136 (− 0.536, 0.264)	0.504	− 0.420 (− 0.717, − 0.124)	0.006*
*Awareness of dental erosion*								
Aware	3 (0.8)	293 (76.1)	56 (14.5)	33 (8.6)	1.188 (0.968, 1.407)	< 0.001*	0.983 (0.796, 1.170)	< 0.001*
Not aware	29 (87.9)	4 (12.1)	0 (0)	0 (0)				

*p < 0.05

^a^Unstandardized B coefficient in the univariate regression model

^b^Unstandardized B coefficient in the multivariate regression model

^c^Age calculated as years in statistical analysis—age ranges presented in the table to reduce the table length

**Fig. 1:**
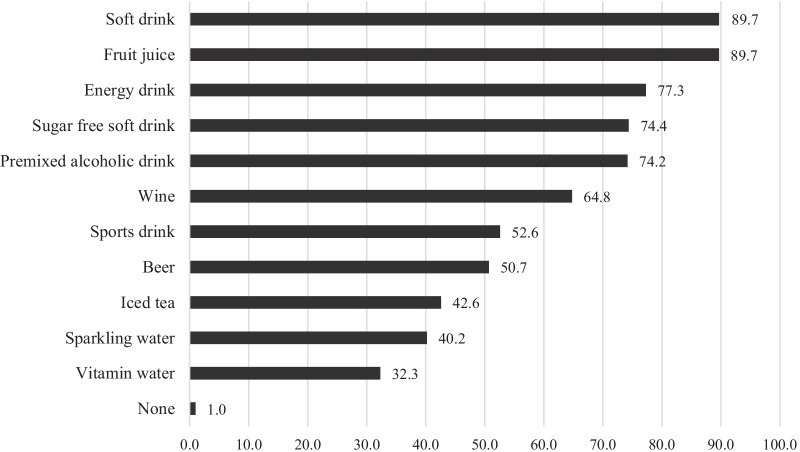
Beverages perceived to be acidic by the percentages of the participants (n = 418)

Soft drink (89.7%) and fruit juice (89.7%) were most often perceived as acidic beverages by the respondents, followed by energy drink (77.3%). Only 1% of the participants failed to identify any acidic beverages (Fig. 1). The ID score for acidic beverages ranged from 0 to 11 points, with a mean of 6.8 ± 2.9 points and a median of 7 points. Table 3 shows the frequency distribution of the ID score in this sample. The ID score was higher in the dentistry (*p* < 0.001, B = 1.563, 95% CI: 0.926, 2.201) and oral health students (*p* < 0.001, B = 2.393, 95% CI: 1.138, 3.647). Other academic fields, age, geographic remoteness, and awareness of dental erosion were not associated with the participant’s ID score (*p* ≥ 0.073).

**Table 3:** Frequency distribution of correctly identified acidic beverages mentioned in Fig. 1, also known as the ID score in this study (n = 418)

	Identified acidic beverages (ID) score [n (%)]											
	0	1	2	3	4	5	6	7	8	9	10	11
*Age in years*^a^												
18–20	0 (0)	5 (5.5)	4 (4.4)	8 (8.8)	7 (7.7)	7 (7.7)	13 (14.3)	14 (15.3)	11 (12.1)	9 (9.9)	7 (7.7)	6 (6.6)
21–30	3 (1.2)	7 (2.8)	11 (4.3)	7 (2.8)	20 (7.9)	33 (13.0)	24 (9.5)	27 (10.7)	26 (10.3)	29 (11.5)	30 (11.8)	36 (14.2)
31–40	1 (2.2)	3 (6.5)	2 (4.4)	0 (0)	4 (8.7)	2 (4.4)	4 (8.7)	6 (13.0)	8 (17.4)	7 (15.2)	3 (6.5)	6 (13.0)
41–72	0 (0)	3 (10.7)	2 (7.1)	7 (25.0)	3 (10.7)	2 (7.1)	0 (0)	2 (7.1)	1 (3.6)	4 (14.3)	2 (7.2)	2 (7.2)
Total	4 (1.0)	18 (4.3)	19 (4.6)	22 (5.3)	34 (8.1)	44 (10.5)	41 (9.8)	49 (11.7)	46 (11.0)	49 (11.7)	42 (10.0)	50 (12.0)

^a^Age calculated as years in statistical analysis—age ranges presented in the table to reduce the table length

### Beverage consumption behaviours

The number of preferred beverages ranged from 0 to 8 types (Table 4), with a mean of 2.1 ± 1.5 types and a median of 2 types. A larger number of preferred beverage types was seen in the students studying paramedicine (*p* = 0.001, B = 0.878, 95% CI: 0.356, 1.400), clinical science (*p* = 0.010, B = 0.709, 95% CI: 0.173, 1.244) or science (*p* = 0.031, B = 0.498, 95% CI: 0.046, 0.950). The number of preferred beverage types was smaller among the students who predominantly resided overseas (*p* = 0.033, B = − 0.921, 95% CI: − 1.767, − 0.075). Other academic fields and geographic areas, age, awareness of dental erosion, knowledge score, and ID score were not related to the number of preferred beverage types (*p* ≥ 0.196).

**Table 4:** Frequency distribution of the number of preferred beverage types in the sample (n = 418)

	Number of preferred beverage types [n (%)]								
	0	1	2	3	4	5	6	7	8
*Age in years*^a^									
18–20	14 (15.4)	17 (18.7)	31 (34.0)	16 (17.6)	4 (4.4)	6 (6.6)	1 (1.1)	2 (2.2)	0 (0)
21–30	33 (13.1)	63 (24.9)	76 (30.0)	45 (17.8)	15 (5.9)	12 (4.7)	6 (2.4)	1 (0.4)	2 (0.8)
31–40	4 (8.7)	13 (28.3)	15 (32.6)	8 (17.4)	2 (4.3)	4 (8.7)	0 (0)	0 (0)	0 (0)
41–72	1 (3.6)	9 (32.1)	6 (21.4)	8 (28.6)	4 (14.3)	0 (0)	0 (0)	0 (0)	0 (0)
Total	52 (12.4)	102 (24.4)	128 (30.6)	77 (18.4)	25 (6.0)	22 (5.3)	7 (1.7)	3 (0.7)	2 (0.5)

^a^Age calculated as years in statistical analysis—age ranges presented in the table to reduce the table length

The quantity of acidic beverage consumption ranged from 0 to 5 cups (250 ml) per day (Table 5), with a mean of 1.4 ± 1.4 cups and a median of 1 cup. The quantity of beverage consumption increased with the age of the participants (*p* < 0.001, B = 0.039, 95% CI: 0.022, 0.055). A higher knowledge score of dental erosion (*p* = 0.001, B = − 0.324, 95% CI: − 0.516, − 0.132) and studying oral health (*p* = 0.011, B = − 0.785, 95% CI: − 1.388, − 0.182) were related to a lower quantity of daily beverage consumption. Other academic fields, geographic remoteness, awareness of dental erosion, and ID score were not associated with the quantity of daily beverage consumption (*p* ≥ 0.092).

**Table 5:** Frequency distribution of the quantity of beverage consumption in the sample (n = 418)

	Cups of beverages consumed per day [n (%)]					
	0	1	2	3	4	5
*Age in years*^a^						
18–20	43 (47.2)	15 (16.5)	20 (22.0)	10 (11.0)	2 (2.2)	1 (1.1)
21–30	103 (40.7)	45 (17.8)	57 (22.5)	29 (11.5)	11 (4.3)	8 (3.2)
31–40	14 (30.4)	8 (17.4)	13 (28.3)	6 (13.0)	1 (2.2)	4 (8.7)
41–72	5 (17.8)	4 (14.3)	7 (25.0)	4 (14.3)	1 (3.6)	7 (25.0)
Total	165 (39.5)	72 (17.2)	97 (23.2)	49 (11.7)	15 (3.6)	20 (4.8)

^a^Age calculated as years in statistical analysis—age ranges presented in the table to reduce the table length

High-risk methods to consume acidic beverages were identified in 191 (45.7%) participants and 227 (54.3%) consumed beverages with low-risk patterns (Table 6). The students that were of greater age (*p* = 0.029, OR = 1.035, 95% CI: 1.004, 1.068), that were studying clinical science (*p* = 0.004, OR = 3.376, 95% CI: 1.473, 7.735), pharmacy (*p* = 0.011, OR = 3.620, 95% CI: 1.337, 9.800), medical science (*p* < 0.001, OR = 5.504, 95% CI: 2.530, 11.973), paramedicine (*p* = 0.013, OR = 2.824, 95% CI: 1.245, 6.409) or science (*p* = 0.001, OR = 3.969, 95% CI: 1.806, 8.722), and that had a higher ID score (*p* = 0.022, OR = 1.091, 95% CI: 1.012, 1.176), were more likely to consume beverages in high risk patterns. Other academic fields, geographic remoteness, awareness of dental erosion, and knowledge score did not have any relationships with the beverage consumption pattern (*p* ≥ 0.086). The Hosmer–Lemeshow test did not find differences between the expected and observed proportions (*p* = 0.385, χ^2^ = 8.510, df = 8) and this indicated a good fit for the logistic regression model.

**Table 6:** Beverage consumption patterns by age, academic fields, geographic remoteness, awareness of dental erosion, knowledge score and ID score (n = 418)

	High-risk pattern [n (%)]	Low-risk pattern [n (%)]	Unadjusted^a^ OR (95% CI)	*p* value	Adjusted^b^ OR (95% CI)	*p* value
*Age in years*^c^						
18–20	31 (34.1)	60 (65.9)	1.059 (1.029, 1.089)	< 0.001*	1.035 (1.004, 1.068)	0.029*
21–30	111 (43.9)	142 (56.1)				
31–40	27 (58.7)	19 (41.3)				
41–72	22 (78.6)	6 (21.4)				
*Academic field*						
Dentistry	31 (29.5)	74 (70.5)	1		1	
Oral Health	5 (23.8)	16 (76.2)	0.746 (0.251, 2.215)	0.598	0.694 (0.232, 2.073)	0.513
Clinical Science	18 (56.3)	14 (43.7)	3.069 (1.359, 6.931)	0.007*	3.376 (1.473, 7.735)	0.004*
Pharmacy	11 (55.0)	9 (45.0)	2.918 (1.100, 7.740)	0.031*	3.620 (1.337, 9.800)	0.011*
Medical Science	38 (71.7)	15 (28.3)	6.047 (2.914, 12.550)	< 0.001*	5.504 (2.530, 11.973)	< 0.001*
Paramedicine	17 (50.0)	17 (50.0)	2.387 (1.081, 5.272)	0.031*	2.824 (1.245, 6.409)	0.013*
Physiotherapy	40 (37.7)	66 (62.3)	1.447 (0.814, 2.570)	0.208	1.688 (0.928, 3.071)	0.086
Science	31 (66.0)	16 (34.0)	4.625 (2.218, 9.643)	< 0.001*	3.969 (1.806, 8.722)	0.001*
*Geographic remoteness*						
Australian major city	71 (43.0)	94 (57.0)	1			
Australian regional and remote	114 (47.3)	127 (52.7)	1.188 (0.798, 1.771)	0.396		0.622
Overseas	6 (50.0)	6 (50.0)	1.324 (0.410, 4.278)	0.639		0.412
*Awareness of dental erosion*						
Aware	176 (45.7)	209 (54.3)	1			
Not aware	15 (45.5)	18 (54.5)	0.990 (0.485, 2.021)	0.977		0.161
*Knowledge score of dental erosion*						
	1.2 ± 0.6^d^	1.3 ± 0.8^d^	0.814 (0.613, 1.080)	0.153		0.104
*ID score*						
	7.0 ± 2.7^d^	6.7 ± 3.1^d^	1.030 (0.965, 1.100)	0.375	1.091 (1.012, 1.176)	0.022*

*p < 0.05

^a^Odds ratio in the univariate regression model

^b^Odds ratio in the multivariate regression model

^c^Age calculated as years in statistical analysis—age ranges presented in the table to reduce the table length

^d^Mean ± standard deviation

## Discussion

### Awareness and knowledge of dental erosion

This study reported a high awareness of dental erosion in Australia, which was at a similar level as in a sample of 18-year-old Norwegians [24]. Although this study adopted a sample of a similar age range as some other researchers have used, the awareness rate here was higher than that reported by them [23, 25, 28]. The dominance of dental and health-related students in this sample might contribute to the high awareness rate. Nevertheless, the science (non-dental and non-health-related) students’ awareness rate in this study was also above 80%. Cross-country differences could be another reason for the discrepancy even though the overseas students included in this sample showed a high awareness level as well. As an improvement in nursing students’ awareness of oral health issues following the implementation of interprofessional education sessions has been reported [39], the effort of interprofessional education made at the university where the survey was conducted could also raise the awareness of dental erosion amongst non-dental students.

Moreover, this study has reported a lower awareness of dental erosion in the adults of greater age. An odds ratio of 0.933 for age means a 6.7% reduction in the likelihood of awareness per additional year of age [38]. Thus, current 30-year-olds were 50% less likely to be aware of dental erosion than current 20-year-olds as 0.933 raised by the power of 10 is approximately equal to 0.500. Even though only 18% of the respondents were older than 30 years of age, the Hosmer–Lemeshow test outcome indicated a good fit for the logistic regression model in likelihood estimation. Table 1 also shows an obvious declination of awareness by age. With a known positive correlation between the age and prevalence of erosive tooth wear in adults [9, 10], to raise the awareness of dental erosion in the mature population is critical. Further, while a stronger awareness did not improve individuals’ ability to identify acidic beverages, as seen in this study, it was associated with the disease-specific knowledge of causes and prevention for dental erosion. Therefore, prevention strategies of erosive tooth wear will benefit by raising the public awareness and knowledge of dental erosion and the role acidic beverages play in its aetiology.

### Beverage consumption

This study also identified an association between the knowledge of dental erosion and quantity of beverage consumption. Our data showed that those adults who demonstrated a higher knowledge score of dental erosion consumed a smaller quantity of beverages per day. This agreed with Skudutyte-Rysstad et al. who have suggested a lack of knowledge is a barrier to the control and prevention of erosive tooth wear [24]. Although the chairside information from the dental team is preferred, a majority of adults have relied on the internet to acquire the knowledge of dental erosion because they have never received the information from dental professionals [40]. Of further note, using the internet and social media could improve health literacy but not the motivation and self-efficacy for health promotion [41]. These indicate the relevance for health care providers including dental practitioners, dietitians, nurses and physicians to offer the evidence-based information of erosive tooth wear to the patients and communities and motivate them to change the behaviour of acidic beverage consumption.

On the other hand, this study found that the individuals demonstrating a greater ability to identify acidic beverages were more likely to consume acidic beverages in high-risk patterns such as drinking between meals or sipping over a long period of time. A recent study has manifested the disconnection between individuals’ capability to differentiate between high and low sugary drinks and their beverage consumption behaviours [42]. As the knowledge of dental erosion and knowledge of acidic beverage identification separately discouraged and enhanced the risk behaviours of dental erosion, the disease-specific and nutrition-specific knowledge might have different effects on health behaviours. Even though the literature has suggested a positive relationship between the level of health knowledge and self-efficacy or adherence to health behaviours [43, 44], it was not uncommon to see behaviours not complying with the health knowledge acquired [45, 46]. Moreover, an article has revealed the influence of parental food behaviour and peer pressure from friends on university students’ unhealthy eating habits [47]. Because health knowledge relies on one’s capacity and motivation in addition to the resources available in the environment [48], health professionals should understand that each individual may have different health knowledge and behaviour. Further investigation to characterise the complexity of beverage consumption behaviours and thereby develop an effective strategy for healthy drinking is also indicated [49, 50].

### Oral health education

Not surprisingly, academic fields made a difference in the awareness and knowledge of dental erosion as well as beverage consumption behaviours in this sample. This was in agreement with a prior work demonstrating “*a knowledge gradient from dental professionals through to healthcare professionals and then to lay members of the community*” [27]. Another study has also found a higher knowledge level of dental erosion in the adults that had a higher level of education [40]. Further, a recent study reported an increase in nursing students’ oral health awareness and knowledge of dental caries upon successful completion of an interprofessional education program at an American university [39]. Whilst it may not be necessary to include oral health knowledge in the programs of other academic fields in order to raise the knowledge level of dental erosion in the public, the effect of education on health literacy is beyond doubt. Nevertheless, owing to the correlations with medicine [17–19], nursing [19, 20], dietetics [21], pharmacy [19], psychology [19, 22], and dentistry and oral health [17–22], erosive tooth wear is a suitable scenario for interprofessional education.

### Limitations

Most of the subjects in this sample were aged between 18 and 34 years and the age distribution due to using a population of university students was a limitation of this study. In addition, this population of interest was composed of the students from the academic fields related to dentistry, health, and science, without the inclusion of other academic backgrounds such as arts, humanities and social science. Using a student population of the targeted programs could have resulted in overestimation of the awareness and knowledge of dental erosion and limited the generalisability of the results. A similar limitation raised from using a total population sampling method to study a population of military clinicians has also been reported [33]. By delivering the questionnaire to a broader population would strengthen the stance on how age influences the awareness and knowledge of dental erosion. With this considered, other variations of the questionnaire may need to be constructed including paper and/or telephone questionnaires to access a wider population.

Traditionally, statistical inference is based on the estimation of sampling errors [51] (p83) and interpretation of probability [51] (p165); and sampling errors become absent when the whole population is studied [51] (p659). This circumstance often leads to concerns about the applicability of statistical inferences drawn from total population studies [52]. Nevertheless, if a study population can be representative of a broader target population which is impossible to sample due to the inclusion of future cases, statistical inference of a total population study is considered generalisable to the target population [52]. The present study used a student population enrolled at an Australian University in 2018. At that time, there was no possibility to include new students enrolled in 2019 and onwards for the wider target population composed of current and future students at the Australian University. From this perspective, inferential statistics of the present study can be generalised to the target population that also includes future students. However, the total and target populations of this study only contain university students, thereby limiting its generalisability to the general population, regardless of the current or future ones.

### Health promotion and future direction

Currently prevention and treatment of erosive tooth wear still focus on the individual actions of the patient and health professionals [11–13], with a lack of health promotion strategies for dental erosion at the government and community levels. The Rethink Sugary Drink campaign in Australia has raised the public awareness of sugar-sweetened beverages, with the interprofessional collaboration among dental hygiene, dentistry, medicine, nutrition, Indigenous health, and community health [53]. The policies to influence sugary drink consumption have also been recommended, such as access and marketing restrictions, pricing strategies, nutrition signposting and labelling, reduction of portion sizes, and reformulation of soft drinks [54]. Similar actions should be taken to prevent beverage acidity from compromising oral health; and these also indicate future research in the effect of integrated health promotion programs on prevention of erosive tooth wear.

## Conclusions

This study has identified an inverse relationship between age and awareness of dental erosion in the sample of Australian University students. In addition, the different effects of disease-specific and nutrition-specific knowledge on beverage consumption behaviours have been manifested by this study. A high awareness rate of dental erosion in this sample has been reported. Within a university setting, the contribution of education to the awareness and knowledge of dental erosion as well as acidic beverage consumption behaviours has also been confirmed.

Erosive tooth wear has become a relevant matter in dentistry, nutrition, and public health. Dental and health professionals should actively provide educational information about dental erosion to the individuals and communities and motivate them to disengage from unhealthy behaviours of acidic beverage consumption. Succeeding investigations into the complexity of beverage consumption behaviours and the development of effective strategies for healthy drinking are indicated.

## Data Availability

The datasets used and/or analysed during the current study are available from the corresponding author on reasonable request.

## Abbreviations

ASGS: Australian Statistical Geography Standard
CI: Confidence interval
df: Degree of freedom
ID score: Identified acidic beverages score
OR: Odds ratio
